# Trifluridine- and tipiracil-induced DPD inhibition mimicking DPD deficiency: a case report

**DOI:** 10.3389/fonc.2025.1591120

**Published:** 2025-08-12

**Authors:** Antonin Schmitt, Baptiste Bouillet, Bernard Royer, François Ghinringhelli

**Affiliations:** ^1^ Université Bourgogne Europe, Centre Georges-François Leclerc, Service Pharmacie, INSERM, CTM UMR 1231, TIRECS, Dijon, France; ^2^ Université Bourgogne Europe, Centre Georges-François Leclerc, Service Oncologie Médicale, INSERM, CTM UMR 1231, TIRECS, Dijon, France; ^3^ Université Bourgogne Europe, INSERM, CTM UMR 1231, TIRECS, Dijon, France; ^4^ Pharmacology and Toxicology Laboratory, Besançon University Hospital, Dijon, France

**Keywords:** trifluridine, tipiracil, DPD deficiency, metastatic colorectal cancer, case report, uracilemia

## Abstract

Fluoropyrimidines, including 5-fluorouracil (5-FU) and its derivatives, remain the standard first-line treatment for metastatic colorectal cancer (mCRC). In recent years, trifluridine/tipiracil (TAS-102), an orally administered combination drug, has become a common third-line therapy for mCRC and could increasingly be used as first-line treatment. We report, for the first time, the case of an mCRC patient presenting discrepancies in uracilemia between measurements taken during (43.0 µg/L) and outside trifluridine/tipiracil treatment (7.3 and 4.5 µg/L). This inconsistency could be attributed to the metabolism of trifluridine into 5-carboxyuracil (5-CU), which can interfere with dihydropyrimidine dehydrogenase (DPD) phenotyping and cause falsely elevated uracilemia. This can lead to unnecessary reduction in the dose of fluoropyrimidines. Clinicians should be aware of this potential interaction when performing DPD phenotyping in patients treated with trifluridine/tipiracil, ensuring that testing is performed either before the treatment begins or after it has finished, or when genotyping *DPYD*.

## Introduction

For patients with unresectable metastatic colorectal cancer (mCRC), 5-fluorouracil (5-FU) or capecitabine remains the backbone of standard first-line treatment in combination with oxaliplatin and/or irinotecan, as well as a targeted biological therapy such as bevacizumab, cetuximab, or panitumumab, unless contraindicated (1). However, therapeutic options are limited after progression in the second line of treatment. European Society of Medical Oncology (ESMO) guidelines state that trifluridine/tipiracil may be a third-line option (2). Median overall survival was improved from 5.3 to 7.1 months as compared to placebo in the pivotal RECOURSE trial (3). Trifluridine/tipiracil (TAS-102) is an orally administered combination drug. It is administered in 28-day cycles, each comprising 5 days of treatment followed by a 2-day rest period for 2 weeks and then a 14-day rest period. Trifluridine is the active compound in the combination, while tipiracil increases its bioavailability.

Trifluridine/tipiracil may also be used as a first-line treatment for patients with dihydropyrimidine dehydrogenase (DPD) deficiency. Indeed, despite its homology with 5-FU, trifluridine is not metabolised by this enzyme and can therefore be safely administered to deficient patients (4). However, these patients should never receive fluoropyrimidine-based treatment due to their enzyme deficiency.

Nevertheless, some patients may be considered ineligible for the usual intensive fluoropyrimidine-based chemotherapy as a first-line treatment due to their age, frailty, altered performance status, or comorbidities. Several recent studies have shown that trifluridine/tipiracil + bevacizumab could be a viable first-line therapy for these patients (5, 6). Nevertheless, these patients may require fluoropyrimidine-based therapies as a second-line treatment and beyond (at least 5-FU monotherapy), which would necessitate DPD phenotyping via uracilemia measurement in some countries, such as France. A threshold uracilemia value set at 16 µg/L helps discriminate between partially deficient patients (uracilemia between 16 and 150 µg/L) and completely deficient patients (uracilemia above 150 µg/L).

Several sources of variability affect uracilemia, including circadian variation, food intake, and sampling conditions (7–9). It has also recently been shown that uracilemia can be falsely increased when measured in patients receiving fluoropyrimidine (10). This is due to the competition between uracil and fluoropyrimidine for DPD. As trifluridine is not metabolised by DPD, an increase in uracilemia in patients receiving trifluridine/tipiracil was not anticipated. Nevertheless, we present, for the first time, the case of a falsely elevated uracilemia in a patient treated with trifluridine/tipiracil.

## Case description

The patient is a 62-year-old married farmer with two children, one of whom still lives with him and his wife. He has no past medical history. His father died of Hodgkin’s disease at the age of 50, and his mother died of uterine cancer at the age of 70. All patient-specific information has been de-identified to protect confidentiality. At the end of 2021, the patient experienced a gradual onset of fatigue, loss of appetite, weight loss, and diarrhoea. Imaging revealed metastatic rectal adenocarcinoma with hepatic and nodal spread. Biopsies confirmed a well-differentiated adenocarcinoma MSS, KRAS, and BRAF wild-type. *DPYD* genotyping was normal, and the patient was *28-homozygous for UGT1A1. Uracilemia in March 2022 was 7.3 µg/L (**Table 1**), which allowed for the initiation of 5-FU treatment. Initial treatment included FOLFOX and panitumumab from April 2022 to December 2023 with no digestive or haematological side effects observed. Only grade 2 neuropathy and skin rash were observed. Following an initial response to treatment, the tumour became resistant to therapy, prompting the initiation of a treatment with FOLFIRI and aflibercept. However, this treatment was frequently interrupted due to digestive side effects (diarrhoea and weight loss). The treatment induced tumour stabilisation until January 2024. A few weeks later, pulmonary, hepatic, and bone progression was observed. The patient was then treated with trifluridine/tipiracil and bevacizumab. Due to disease progression and the use of bevacizumab, rectal bleeding required haemostatic radiotherapy. As 5-FU re-administration was being considered while the patient was receiving trifluridine/tipiracil and bevacizumab, uracil concentration was measured at 43.0 µg/L on December 5, 2024 (**Table 1**). It was therefore decided to switch to regorafenib. Fifteen days after stopping trifluridine/tipiracil and bevacizumab, just before starting regorafenib, uracilemia was tested again due to the discrepancy between two measurements and found to be 4.5 µg/L.

**Table 1:** Uracilemia values.

	March 30, 2022	December 5, 2024	December 20, 2024
Uracilemia (µg/L)	7.3	43.0	4.5
Glomerular filtration rate (CKD-EPI; mL/min/1.73 m²)	94	110	102

## Patient perspective

The patient died on January 26, 2025, following cardiorespiratory arrest due to haemorrhagic shock linked to satellite rectal bleeding from his metastatic rectal tumour. The patient’s perspectives were therefore not collected.

## Discussion

DPD activity should be evaluated before any fluoropyrimidine administration. This can be achieved through *DPYD* genotyping, which enables the early detection of genetic variants that predispose patients to severe toxicity. However, genotype testing may miss rare or unknown mutations in the *DPYD* gene and does not fully predict the severity of toxicity, as other genetic or environmental factors can influence the clinical outcome. Although unsuitable for daily care, the gold standard approach consists of measuring DPD activity in peripheral blood mononuclear cells (PBMCs) (11). Phenotyping by uracil concentration measurement is the method approved by the French health authorities [Institut du Cancer/Haute Autorité de Santé (INCa/HAS)]. Uracilemia is sensitive to several factors, the most important of which are pre-analytical and analytical issues (9), sampling while patients are being treated with fluoropyrimidines (10), and renal insufficiency (12, 13). All of these factors were either controlled or within the range in this patient. Other less significant sources of variability are tumour lysis syndrome (14), a high-meat diet (8), and the nycthemeral cycle (9). They were not all controlled in our case, but they have a limited impact. Thus, none of these sources of variability could explain the differences in uracil measurements observed in our patient.

Trifluridine/tipiracil is a well-known and useful option for patients with DPD deficiency since the main active compound (trifluridine) is a fluorinated pyrimidine that, like 5-FU, disrupts DNA. However, unlike 5-FU, trifluridine is not metabolised by DPD. Nevertheless, a small amount of trifluridine is metabolised into 5-carboxyuracil (5-CU) (15). 5-CU can be decarboxylated by iso-orotate decarboxylase (an enzyme of the thymidine salvage pathway) to form uracil, which could possibly explain the increase in uracil levels during treatment with trifluridine/tipiracil (16). Although 5-CU levels in plasma are low, it appears to be sufficient to increase uracilemia at least up to almost 35–40 µg/L. Unfortunately, no 5-CU concentrations were available in this case.

Thus, despite the lack of risk of using trifluridine/tipiracil in DPD-deficient patients, clinicians need to be aware of the potential interaction between the trifluridine metabolite and DPD phenotyping, as it may falsely give a deficient result if the patient is treated with trifluridine/tipiracil at the time of uracil measurement. Therefore, if DPD phenotyping is to be performed, it should always be performed at the time of mCRC diagnosis or at least at an extended period after any intake of fluoropyrimidine or trifluridine/tipiracil. Genotyping of *DPYD* could alternatively be preferred in such situations.

## Data Availability

The original contributions presented in the study are included in the article/supplementary material. Further inquiries can be directed to the corresponding author.
